# Differences in primary metabolism related to quality of raspberry (*Rubus idaeus* L.) fruit under open field and protected soilless culture growing conditions

**DOI:** 10.3389/fpls.2023.1324066

**Published:** 2024-01-11

**Authors:** Claudia Fuentealba, Fernanda Álvarez, Excequel Ponce, Sebastian Veas, Martina Salazar, Diego Romero, Anibal Ayala-Raso, Juan E. Alvaro, Monika Valdenegro, Carlos R. Figueroa, Lida Fuentes

**Affiliations:** ^1^ Escuela de Agronomía, Facultad de Ciencias Agronómicas y de los Alimentos, Pontificia Universidad Católica de Valparaíso, Quillota, Chile; ^2^ Centro Regional de Estudios en Alimentos Saludables (CREAS), Valparaíso, Chile; ^3^ Instituto de Estadística, Facultad de Ciencias, Universidad de Valparaíso, Valparaíso, Chile; ^4^ Laboratory of Plant Molecular Physiology, Institute of Biological Sciences, Universidad de Talca, Talca, Chile

**Keywords:** metabolic pathways, fruit quality, sugars, softening, soilless culture

## Abstract

**Introduction:**

The raspberry (*Rubus idaeus*) fruit is characterized by good taste and high acceptability by consumers. Thus, the impact on the quality attributes and metabolites related to raspberry taste should be evaluated in crop alternatives such as the protected soilless culture. This study aimed to evaluate the metabolic changes during fruit development and postharvest of raspberry grown in open field and protected soilless culture and their relationship with quality parameters and sensory perception.

**Methods:**

In this study, the quality parameters and polar metabolites -sugar and amino acids- content were evaluated during raspberry ripening. In addition, ripe fruit was stored at 1 °C for five days, followed by one day of shelf life at 20 °C.

**Results:**

The physiological and quality parameters showed typical changes during ripening in both growing conditions: a constant production of CO_2_, a drastic loss of firmness, an increase in weight and soluble solids content, loss of acidity, and a turning to red color from the green to fully ripe fruit stages in both growing conditions. Fruit from the protected soilless culture had significantly higher weight but a lower soluble solids content. The metabolic analysis showed differences in primary metabolites content during ripening and storage at 1 °C between both growing conditions. The raspberries grown in the open field showed higher contents of sugars such as D-glucose and D-fructose. On the contrary, the fruit from the protected soilless culture showed higher contents of some amino acids such as L-alanine, L-serine and L-valine, among others. The sensorial panel showed significant differences in the perception of the sweetness, acidity, color and firmness of ripe fruit from both growing conditions.

**Discussion:**

The present study provides interesting and useful results with direct commercial application for this alternative growing system, mainly in areas where soil and water scarcity are a reality.

## 1 Introduction

Raspberries (*Rubus idaeus* L.) have an attractive color, unique flavor, and valuable health benefits. These include being a valuable source of vitamins, minerals, dietary fiber, and antioxidant compounds (Graham and Simpson, 2018; Popović et al., 2022). During raspberry ripening, a progressive decrease in fruit firmness can be observed, associated with cell wall modification and increased sugar composition in the ripe fruit (Stewart et al., 2001; Vicente et al., 2007; Graham and Simpson, 2018). It has been reported that the onset of ripening is characterized by the accumulation of organic acids and soluble sugars, which contribute to acquiring the fleshy trait associated with cell expansion (Dincheva et al., 2013). Once the fruit ripens, it reaches approximately 9% of soluble solids content, of which 5%–6% correspond to sugars (glucose, fructose, and a smaller amount of sucrose) (Graham and Simpson, 2018). In addition, the quality and marketability of raspberry is highly influenced by the concentration of fructose, glucose, and sucrose as main soluble sugars (Kafkas et al., 2008; Dincheva et al., 2013).

The composition of fruits can depend on variety, maturity stage, and environmental and cultivation conditions (Kassim et al., 2009; Akhatou et al., 2016; Marchi et al., 2019; Akšić et al., 2022; Kotuła et al., 2022). Raspberries grown by conventional and organic cultivation have been studied to evaluate the yield, quality, and phytochemical composition of the plant and its fruits (Anjos et al., 2020; Frías-Moreno et al., 2021; Kotuła et al., 2022). Moreover, covered tunnels and greenhouse soilless culture have become an attractive alternative in places with scarce soil and water. In addition, raspberries for dessert are primarily cultivated in fertigated soilless culture under cover (Stojanov et al., 2019; Balawejder et al., 2023). Pritts et al. (1999) reported that ripe raspberry fruit produced in greenhouse conditions were larger, firmer, and much less prone to fruit rot than open field production. Even more recently, Marchi et al. (2019) revealed that the yield of raspberry fruit grown under plastic cover in Brazil was higher than non-covered but depends greatly on the cultivar and the harvesting productive cycle (fall or spring). In addition, Balawejder et al. (2023) improved the synthesis of bioactive compounds through substrate modification in the soilless cultivation of raspberries. Likewise, the primary metabolites in strawberries, such as sugar and amino and organic acids, varied when they were cultivated under the macro tunnel, different substrates, and electrical conductivities of irrigation (Akhatou et al., 2016). In summary, quality parameters are subject to certain preharvest process or factors that could be improved. These processes are as follows: the optimization of the interactions between cultivars, environmental conditions (temperature, light, concentration of CO_2_, and vapor pressure deficit (VPD)) and the composition and concentration of nutrient solutions, crop management, growing media together with the electrical conductivity threshold value. The use of adapting fertigation to growing conditions and/or nutrient solutions of moderately high conductivity seems promising in providing high yields of remarkable quality while reducing the emission of nutrients to the environment in greenhouses (Moya et al., 2017; Rodríguez et al., 2019). However, to our knowledge, no studies associate the changes in raspberry fruit quality and its acceptability with metabolic changes during ripening in protected soilless culture and open field.

The metabolomic profile has been an innovative tool for understanding the metabolic response of plants exposed to different growing and storage conditions. Ren et al. (2023) used widely targeted metabolome of raspberries to correlate the antioxidant capacity and metabolites of fruit grown under different environments. Similarly, Durán-Soria et al. (2021) used a multi-platform metabolomic approach to contrast the metabolite profiles associated with flavor and nutrition in different raspberry cultivars. This study aimed to determine the change in the quality and polar metabolites profile during the ripening and storage of raspberry grown in the open field and protected soilless culture growing conditions.

## 2 Materials and methods

### 2.1 Plant material

Raspberry (*R. idaeus* ‘Heritage’) fruit was collected from commercial orchards located in Casablanca (longitude 33°20′39ʺS; latitude 71°22′07ʺW; 247 m.a.s.l.), Chile, and greenhouse located at the experimental station (Facultad de Ciencias Agronómicas y de los Alimentos, Pontificia Universidad Católica de Valparaíso, located in Quillota (longitude 32°53′43ʺS; latitude 71°12′25ʺW, 120 m.a.s.l). The protected soilless growing condition was in a greenhouse (polyethylene cover thickness, 200 μm) with a natural ventilation system. The unit crop was a 10.5-L Projar Golden Grow Blend Medium Washed (Valencia, Spain) coir growth pot (235 × 235 mm, H × W) with one bare root raspberry cane transplanted from the nursery at an approximate age of 3 months. The distance between the unit crop corresponded to 0.4 m and between the rows to 1.6 m. Each unit crop was fertigated by one pressure compensated dripper, with a nominal flowrate of 4.0 L·h^−1^ and with four manifolds and their corresponding microtubes and spikes. The raspberry plants were fertigated with a standard nutrient solution adjusted to specific requirements and daily monitored during the research. For the trial, two checkpoints were used for fertigation control: 1) a control dripper (4.0 L h^−1^) and 2) a drain pan to monitor the supplied fertigation and its absorption response. The pH, volume of nutrient solution, and EC of the fertigation input and drainage were measured at the control points. The nutrient solution EC was maintained at 1.7 mS cm^−1^ ± 0.1 and pH at 5.8 ± 0.1. Each fertigation pulse was activated when 10% of the readily available water in the substrate had been used and the volume necessary to produce between 20% and 30% drainage to avoid any accumulation of salts. Pruning and other cultural management practices were performed according to commercial practices.

A commercial orchard with traditional growing conditions was selected for fruit grown in open field conditions. The distance between the unit crop corresponded to 1 m and between the rows to 2 m. The plants were watered for 2 h every 3 days by flooding irrigation. During harvest time, the fertilization doses used were of 76, 76, 38, 19, 19, and 1 kg ha^−1^ year^−1^ for the nitrogen, potassium, phosphorus, sulfur, magnesium, and calcium elements and 1.9 kg ha^−1^ year^−1^ for the boron element, respectively (Hirzel, 2013). The Casablanca soil is classified as kaolinitic, fine, thermic Ultic Palexeralf (Cauquenes Series), derived from granitic materials, subangular blocky structure, clay loam texture, reddish brown (5YR4/4), slope 11.5%, moderate permeability and drainage, and rapid runoff (Quezada et al., 2014).

The average minimum and maximum temperature for open field (Casablanca) was downloaded from Red Agrometereológica INIA data (available at: https://agrometeorologia.cl/). On the other hand, the average temperature and humidity for the protected culture were registered using a HOBO Pro v2 Temperature/Relative Humidity Recorder.

The fruits from both growing conditions were harvested in January 2022. The experimental design consisted of four blocks of six plants for each growing condition. For quality parameters, fruits attached to the receptacle and with peduncle were harvested and sorted by color and size as large green (LG), white (W), pink (P), red or ripe (R), and over ripe (OR) stages (Monsalve et al., 2022; Álvarez et al., 2023). Four independent experimental units of 20 intact fruits attached to the receptacle per developmental stages were used for quality assessments. Immediately after harvest, half of the collected fruit was frozen in liquid nitrogen for metabolite extraction. The other half was used to determine the CO_2_ production and quality parameters. In addition, a sensory panel was performed with ripe fruits (R stage).

Ripe fruits from each growing condition were detached from the receptacle and used for the postharvest assay. A total of 12 experimental units with four fruits (three units per block) were used per each growing condition. The fruits were evaluated at harvest (Day 0), after storage for 5 days at 1°C when the fruits were taken out of cold storage [Day 5 (1°C)], and after cold storage and 1 day of shelf life at 20°C [Day 5 (1°C) + 1 (20°C)].

### 2.2 CO_2_ production and fruit quality assessment

The CO_2_ determination was performed according to Monsalve et al. (2022). The samples of each independent unit for each assay were introduced into close tight chambers (390 mL) and were incubated at 20°C for 2 h. The needle of a CO_2_ detector (MAP Headspace gas analyzer, Bridge Analyzers, USA) was injected into the chambers, and the CO_2_ concentrations were recorded. The results were expressed as milligrams of CO_2_ per kilogram per hour.

The individual weight of each fruit from the experimental units of developmental stages and postharvest assay per growing condition was measured using an analytical balance. The moisture of ripe fruit was determinate according to AOAC protocols (AOAC (Association of Official Analytical Chemists), 1997). The color of drupelets was determined in each fruit per developmental stage and after postharvest. The color was recorded using a chroma meter coupled with a Minolta DP-301 data processor (model CR-300, Konica Minolta; Tokyo, Japan). The measure was detailed as the CIELAB scale (L*, a*, b*, hue angle-h°, and chroma-C). Fruit firmness was determined as the force necessary to deform the fruit in 1 mm using the FirmPro equipment (Happy Volt SPA, Santiago, Chile) and expressed as Newton (N) (Monsalve et al., 2022). Six grams of drupelets from each replicate were homogenized in a mortar. The juice was analyzed for total soluble solids (TSS) using a refractometer (ATAGO, Tokyo, Japan), expressed as total soluble solid content TSS (brix degree), and the titratable acidity (TA) was analyzed using a pH meter (model PL-500, Ezdo; Taipei, Taiwan) and expressed as the percentage of citric acid per 100 g of fresh weight (FW) of fruit.

### 2.3 Untargeted metabolomic analysis

The primary metabolites were evaluated at the W, P, and R stages of development and during the postharvest trials. Extraction and derivatization of polar metabolites were performed according to Fuentealba et al. (2017). A total of 100 mg of frozen drupelets powder was mixed with 500 µL of cold methanol and 20 µL of 3 mg mL^−1^ phenyl β-D-glucopyranoside was used as internal standard. The determination was done using an Agilent 7890B gas chromatograph equipped with an HP-5 ms Ultra Inert column (30 m × 0.25 mm × 0.25 µm), a 5977A single quadrupole mass detector and an electron impact ionization source (GC-MS) (Agilent Technologies, Santa Clara, CA, USA). Chromatographic peaks were deconvoluted and identified by comparing retention times and mass spectra with a library constructed from commercial standards and the NIST14 library using Mass Hunter Quantitative software (Agilent Technologies, Santa Clara, CA, USA). The data were expressed as relative abundance, which was obtained dividing the peak area of each compound by the peak area of the internal standard (phenyl β-D-glucopyranoside), the fresh sample weight, and quality control (QC) that consisted of a mixture of all samples.

### 2.4 Sensory determinations

The sensory differences in sweetness, acidity, firmness, and color of ripe fruit from protected soilless culture and open field were determined in “Centro Regional de Estudios de Alimentos Saludables-CREAS” (Valparaiso, Chile). A total of 20 panelist was trained with increased concentration of sucrose solutions (4, 6, 9, and 12 g L^−1^) and a fixed concentration of citric acid (0.43 g L^−1^) according to PN-ISO 3972:2016–07 normative (2016). After four training sessions, 10 panelists (30 years’ average age), five women and five men, were chosen for the sensory determinations on raspberry. First, a duo–trio test (Mihafu et al., 2020) was carried out using fruit from open field conditions as the reference, and one sample from the open field and another from protected soilless growing conditions were used as coded samples. The panelists were asked to select the coded sample with the same sensorial perception of the reference. Then, the sweetness discrimination of ripe fruit from protected soilless culture and open field was done by a sensorial paired comparison test without a reference sample. A pulp from a mix of fruits from the respective growing conditions was used as the sample for the panelists to evaluate sweetness and acidity, and fruit was used to evaluate firmness and color. The tests were carried out in three sessions for each panelist. The results were expressed as the number of positive or negative hits per attribute.

### 2.5 Statistical analysis

The physiological and quality data for each growing condition were analyzed using the ANOVA test and visualized using GraphPad Prism 8.0.2 (GraphPad Software, San Diego, CA, USA). During fruit development and postharvest, asterisks indicate significant differences in physiological and quality parameters between ripening stages with LG stage, *p* ≤ 0.05 (*), *p* ≤ 0.01 (**), *p* ≤ 0.001 (***), and *p* ≤ 0.0001 (****). Metabolomics data and correlation of response variables were analyzed by principal component analysis (PCA) and partial least squares discriminant analysis (PLS-DA) using MetaboAnalyst 5.0 (https://www.metaboanalyst.ca). The metabolites were associated with the metabolic pathways using MetaboAnalyst 5.0. The correlation between metabolite compounds was carried out by the Pearson method. The criteria selection was with a *p*-value < 0.05 and an FDR value < 0.5. During the sensory evaluation, the data obtained by the duo–trio and paired test was evaluated by t-test. The differences were established for α = 0.05 (*) considering seven hits of 10 answers and α = 0.01 (**) considering eight hits of 10 answers in the three sessions, respectively (ISO, 2005; Iborra-Bernad et al., 2014).

## 3 Results

### 3.1 Fruit quality during ripening and postharvest

**Figure 1:**
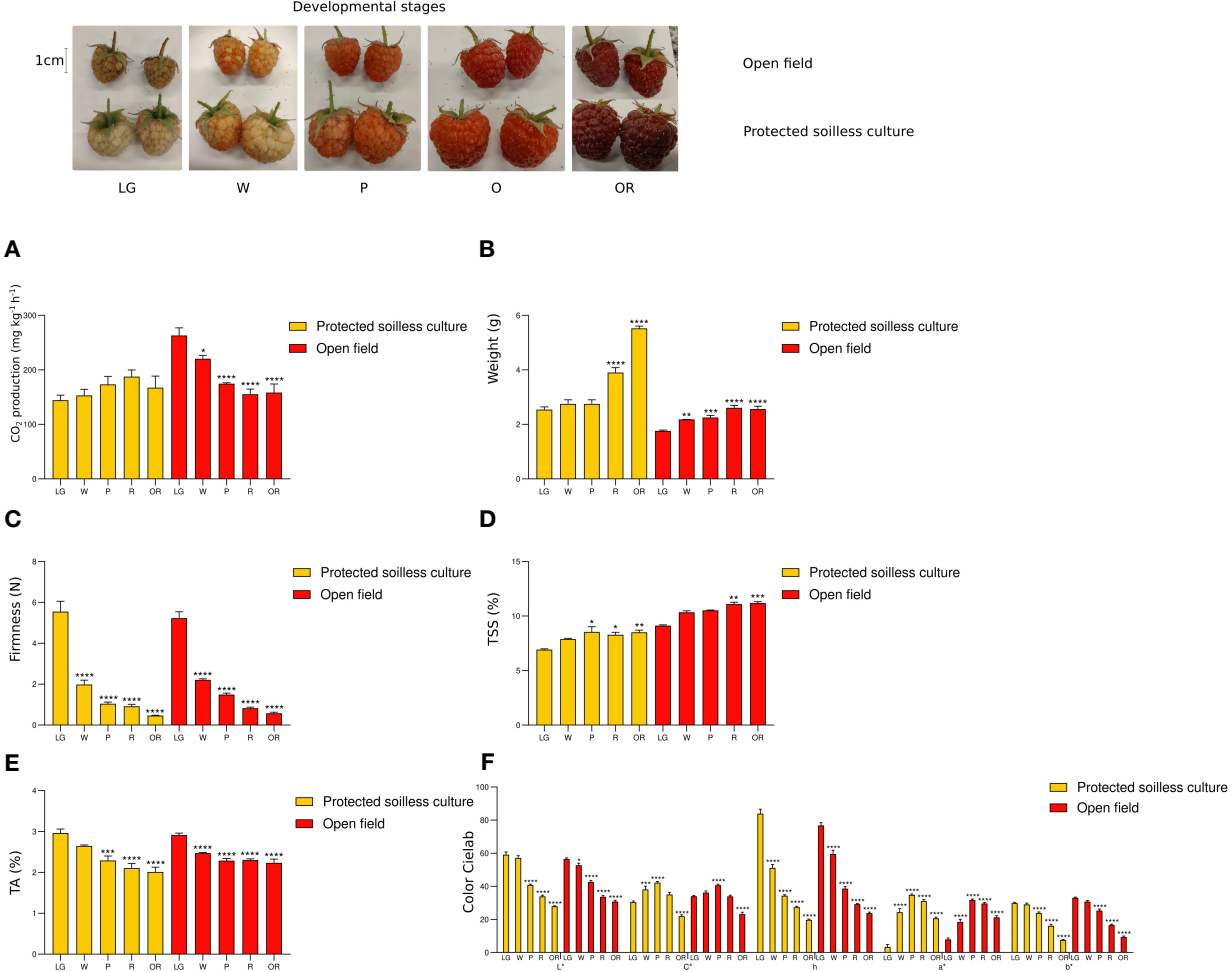
Physiological and quality parameters during raspberry fruit development. Measurements were performed in fruits from protected soilless culture and open field growing conditions. The upper panel shows the different fruit developmental stages in both seasons (bar = 1 cm). LG, large green; W, white; P, pink; R, ripe fruit; and OR, overripe fruit stages. CO_2_ production (mg CO2 kg^−1^ h^−1^; **A**), weight (g; **B**), firmness (Newtons; **C**) and color (Cielab scale; **F**) were determined in whole fruit. Total soluble solids content (TSS, °Brix; **D**) and titratable acidity (TA, %; **E**) were determined in drupelets. Data represent the means ± S.D. from four sample units (each containing 20 fruits) in each developmental stage. Asterisks indicate significant differences of physiological and quality parameters between ripening stages with LG stage, *p* ≤ 0.05 (*), *p* ≤ 0.01 (**), *p* ≤ 0.001 (***), and *p* ≤ 0.0001 (****).

The ripening stages (**Figure 1**) of raspberries harvested in open field and protected soilless culture growing conditions were characterized by the evolution in CO_2_ production and quality parameters. A constant CO_2_ production was observed during ripening in protected soilless growing conditions, and a significant decrease in CO_2_ production during ripening was observed in open field growing conditions (**Figure 1A**). The weight was the most remarkable difference between fruit harvested under open field and protected soilless culture growing conditions. Even though the weight increased significantly in both growing conditions, the pattern of weight gain showed differences in the fruit of the protected soilless condition. The ripe fruits from the protected soilless culture showed higher weight in the OR stage (3.9 ± 0.4 g) compared to those grown in the open field (2.6 ± 0.2 g), and the fruit from the protected soilless culture continued to gain weight after the white stage (**Figure 1B**). On the other hand, a similar trend in color parameters was observed in both growing conditions, with a significant decrease in brightness (L*), hue angle-h°, and b*, and a significant increase in red color (a*) parameter from the large green (LG) stage to the pink (P) stage during ripening (**Figure 1F**).

In both growing conditions, firmness half decreased from the LG to the W stages (**Figure 1C**). Although the average firmness for both conditions was similar (protected soilless, 0.9 ± 0.3 N; open field, 0.82 ± 0.2 N), fruit with greater firmness was recorded in greenhouse conditions (1.5N) compared to field conditions (1.1N). A significant increase in total soluble solids content (TSS) was observed from the W stages for protected soilless culture and the R stage for open field, with a higher TSS content in the ripe fruit of the open field growing (11.1 ± 0.4 Brix degree) compared to protected soilless condition (8.3 ± 0.5 Brix degree) (**Figure 1D**). The moisture observed was 83.15% ± 2.79% and 85.17% ± 0.99% in the ripe fruit of protected soilless and open field conditions, respectively, suggesting that TSS do not vary due to changes in humidity.

A significant decrease in titratable acidity (TA) was observed in fruit from the LG to P stages of the protected culture and from the LG to W stages of the open field growing condition (**Figure 1E**) and similar acidity for the ripe fruit from both growing conditions (protected condition: 2.1% ± 0.3%; open field condition: 2.3% ± 0.1%).

**Figure 2:**
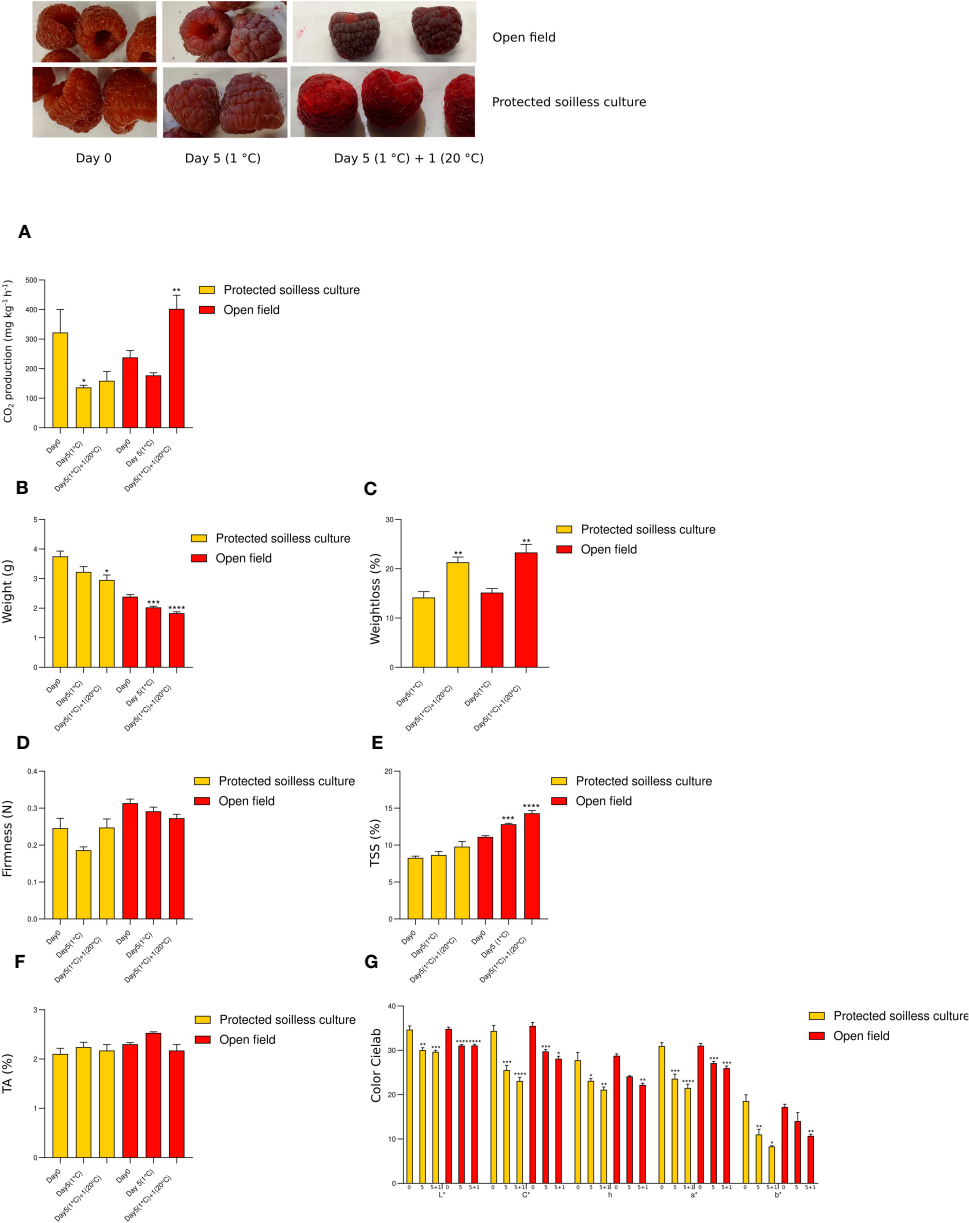
Physiological and quality parameters during raspberry postharvest. Measurements were performed in fruits from protected soilless culture and open field growing conditions. CO_2_ production (mg CO_2_ kg^−1^ h^−1^; **A**), weight and weight loss (g; **B**; **C**), firmness (Newtons; **D**), and color (Cielab scale; **G**) were determined in whole fruit. Total soluble solids content (TSS, °Brix; **E**) and titratable acidity (TA, %; **F**) were determined in drupelets. Data represent the means ± S.D. from five sample units (each containing ten fruits) in each developmental stage. Asterisks indicate significant differences of physiological and quality parameters between ripening stages with LG stage, *P* ≤ 0.05 (*), *P* ≤ 0.01 (**), *P* ≤ 0.001 (***), and *P* ≤ 0.0001 (****).

During the postharvest of ripe fruit (**Figure 2**), different CO_2_ production patterns were observed for the fruit of each growing condition, with a significant increase after cold storage [Day 5 (1°C)] and shelf life [Day 5 (1°C) + 1 (20°C)] of raspberries harvested in the open field compared to harvest day (**Figure 2A**). The decrease in weight of open field fruits during postharvest compared to fresh ripe fruit was highly significant, unlike protected soilless fruit, whose weight does not vary significantly during storage at 1°C (**Figure 2B**). The fruits from both growing conditions showed a significant weight decrease after storage at 1°C, and the weight loss significantly increased after the shelf life of fruit from both growing conditions (**Figure 2C**). After storage at 1°C, the weight loss was nearly 15% and increased significantly to 20% after shelf life at 20°C for fruits from both growing conditions. The firmness showed no significant decrease after cold storage, and the shelf life at 20°C for fruit from protected soilless culture and open field was similar (**Figure 2D**). The TSS content was significantly higher in open field growing conditions after cold storage and shelf life, without significant differences in the fruit from the protected culture (**Figure 2E**). The TA showed no significant differences during postharvest (**Figure 2F**). A significant decrease in the brightness (L*) and red color (a*) of fruit from both growing conditions was observed during postharvest, the loss of brightness of the ripe fruit grown in the open field being higher (**Figure 2G**).

### 3.2 Primary metabolites differences between soilless culture and open field harvest fruit

**Figure 3:**
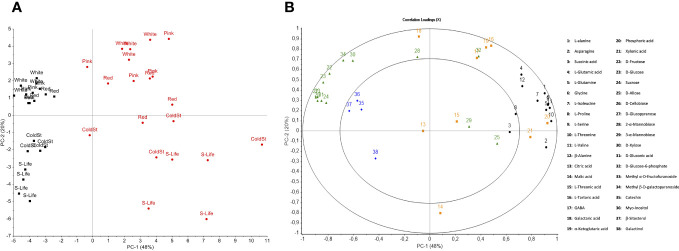
Principal component analysis (PCA) of the metabolic profile of raspberries. **(A)** Scores plot. Raspberries grown in protected soilless culture are represented in red and fruit grown in open field are represented in black. The different pre- and postharvest stages are represented as: White; Pink; Ripe fruit; ColdSt: cold storage (5 days at 1°C); and S-life: shelf life (5 days at 1°C and 1 day at 20°C). **(B)** Loadings plot. The amino acids are represented in black, organic acids in orange, sugars in green, and other compounds in blue.

**Figure 4:**
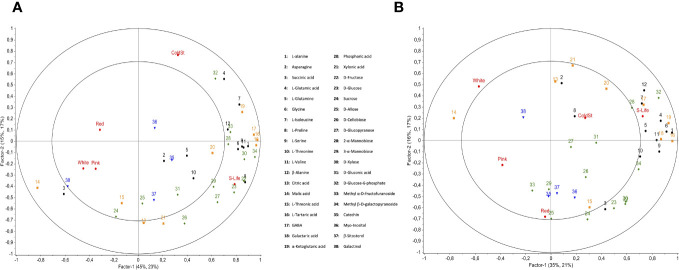
Partial least squares discriminant analysis (PLS-DA) of polar metabolites for raspberries grown in open field **(A)** and grown in protected soilless culture **(B)**. Red dots correspond to the development stages (White, Pink, and Ripe fruit) and postharvest time (ColdSt: cold storage (5 days at 1°C); and S-life: shelf life (5 days at 1°C and 1 day at 20°C). The amino acids are represented in black, organic acids in orange, sugars in green, and other compounds in blue.

For the metabolomic analysis, PCA and PLS-DA were performed (**Figures 3**, **4**). The relative abundance of untargeted metabolites was used as response variables. The PCA showed that the first two components (PC1 and PC2) explain 68% of the variability determined by all data analyzed, where the PC1 discriminates between growing conditions and PC2 between ripening stages and postharvest sampling (**Figure 3A**). When evaluating scores and loadings plots together, a higher concentration of sugars, including the main ones such as D-glucose, D-fructose, and sucrose, were observed in the raspberries from the open field and (left side of both graphs in **Figure 3**). On the other hand, the samples from the protected soilless culture showed higher concentrations of all the amino acids detected (right side of both graphs in **Figure 3**). During fruit development and ripe fruit postharvest, a positive correlation was observed between sucrose and D-fructose (0.87), sucrose and D-glucose (0.89), and D-fructose and D-glucose (0.99) (**Supplementary Figure 1**).

**Figure 5:**
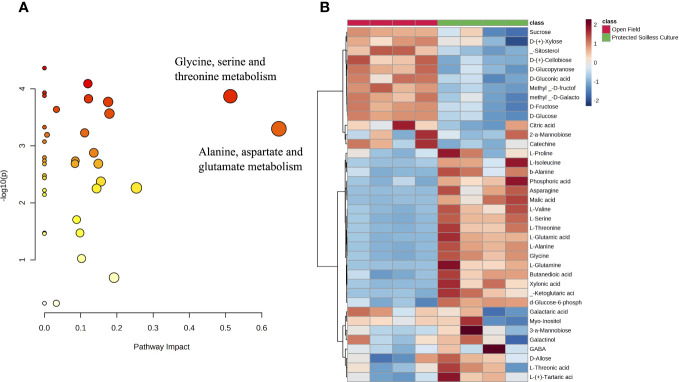
Metabolite pathway analysis during raspberry shelf life. **(A)** Pathways impact analysis. The metabolic pathways are depicted as circles based on their enrichment scores (y-axis) and topology analyses (pathway impact, x-axis) conducted with MetaboAnalyst 5.0. The intensity of the circle colors (from yellow to red) indicates the extent of significant metabolite changes in each respective pathway. **(B)** Hierarchical clustering analysis heatmap performed using all metabolites during raspberries shelf life [Day 5 (1°C) + 1 (20°C)] between grown in the open field and protected soilless culture.

Two independent PLS-DAs of each growing condition were conducted to observe the metabolic changes during ripening and fruit postharvest (**Figure 4**). Both growing conditions presented high malic acid and galactinol contents at white and pink stages. The main difference between the open field (**Figure 4A**) and protected soilless culture (**Figure 4B**) was found in shelf-life, where the fruit from the protected soilless culture correlated to amino acids and those from the open field to sugars. Furthermore, the analysis of metabolic pathways revealed significant differences in metabolites involved in the aspartate, alanine, and glutamate metabolism, and glycine, serine, and threonine metabolism during shelf life (**Figure 5**). On the other hand, at the pink stage, the metabolites that showed differences were D-gluconic acid and glucose-6-phosphate, which are related to the pentose phosphate pathway and were more abundant in fruit grown in open field (data not shown).

### 3.3 Sensorial differences between protected soilless culture and open field harvest fruit

**Figure 6:**
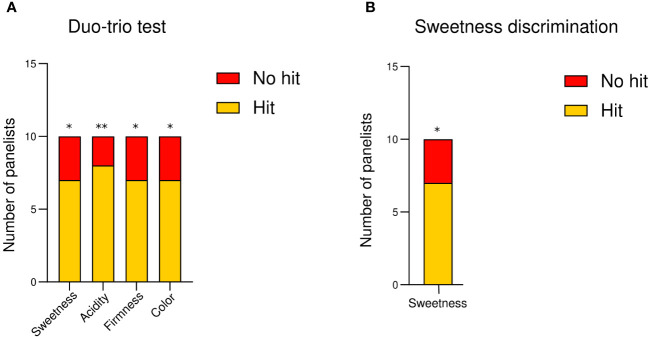
The number of the success’s sensorial sample discrimination. **(A)** The sensory differences in sweetness, acidity, firmness, and color of ripe fruit from protected soilless culture and open field were determined a duo–trio test using fruit from open field conditions as the reference, and samples from each growing conditions as coded samples. **(B)** Then, the sweetness discrimination of ripe fruit from protected soilless culture and open field was done by a sensorial paired comparison test without a reference sample. A pulp from a mix of fruits was to evaluate sweetness and acidity, and intact fruits were used to evaluate firmness and color. The tests were carried out in three sessions for each panelist. The results were expressed as the number of positive or negative hits per attribute. The differences were established for α = 0.05 (*) considering seven hits of ten answers, α = 0.01 (**) considering eight hits of 10 answers, respectively.

The sensory differences in ripe fruit from protected soilless culture and open field determined by a duo–trio test showed an average of seven correct answers from the 10 panelists to correctly identify the open field samples in sweetness, firmness, and color, differentiating them from the greenhouse samples. Regarding acidity, eight correct answers were recorded. The sensorial paired comparison test without a reference sample to determine the sweetness sample showed an average of the 10 panelists could distinguish differences in the sweetness of raspberries (**Figure 6B**). According to the statistical analyses, all sensory parameters show significant differences. Therefore, the significant differences in the total soluble solids content (**Figure 1D**) and at the level of primary metabolites (**Figure 4**) were discriminated in the sensory panel.

## 4 Discussion

In the present research, we showed quality and metabolomic changes in raspberries growing in two different conditions (i.e., greenhouse and open field) during fruit development and postharvest. Here, we measured CO_2_ production, fruit quality parameters, primary metabolites, and sensory determinations in both cultivation conditions.

Previous studies in different raspberry cultivars, including ‘Heritage’, showed a variable trend of CO_2_ production of the fruit, although it coincides with the non-climacteric fruit behavior (Perkins-Veazie and Nonnecke, 1992; Contreras et al., 2021; Monsalve et al., 2022). In the present study, both cultivation conditions present a differential CO_2_ production during ripening. These differences in CO_2_ production patterns are probably directly related to temperature, since differences in CO_2_ production have been observed for Heritage cultivar in the same location in a different season (Fuentes et al., 2015; Bernales et al., 2019).

During the fruit development of ‘Heritage’ raspberry, an increased fruit weight and size from the small green to the white stages has been reported. Then, when fruits reached 2 g average at the white stage, the weight increase was less pronounced from the white to the ripe stages (Bernales et al., 2019). In the present study, fruits grown in the protected soilless culture had a significantly higher weight than those grown in the open field, and the fruits grown in the protected soilless culture continued to gain weight after the white stage (**Figure 1B**). The average weight obtained for the fruits at the mature (R) stage grown in the greenhouse was 4 g, a value higher than what was previously reported (2.8 g) in the Heritage cultivar under open field growing conditions (Bernales et al., 2019). In addition to the higher weight that the ripe fruit achieves growing in protected conditions, it is interesting to highlight that the weight of this fruit does not vary significantly during storage at 1°C (**Figure 2B**). In tomato, it has been reported that fruit weight can be modified by the value of the electrical conductivity of the nutrient solution (Moya et al., 2017). These results suggest that the major weight of raspberry fruit grown in the protected soilless culture could be by nutrient solution (1.7 mS cm^−1^). However, strawberries grown in soilless conditions with different electrical conductivity levels (1.5, 2.5, 3.5, and 4.5 mS cm^−1^) showed the highest fruit average weight at 2.5 mS cm^−1^, which was not dependent on the increased electrical conductivity (D'Anna et al., 2003). Therefore, the effect of electrical conductivity on the size of raspberry fruits and the weight loss during fruit postharvest should be further studied.

Independent of raspberry cultivar, ripening is associated with a decrease in fruit firmness and increased sugar composition (Stewart et al., 2001; Vicente et al., 2007; Graham and Simpson, 2018; Contreras et al., 2021; Monsalve et al., 2022; Álvarez et al., 2023). In the present study, a decrease in firmness and titratable acidity was observed during ripening, with similar average values at the same developmental stage of both growing conditions. Despite the average firmness being similar at the ripe stage and during postharvest for both conditions, fruit grown in soilless conditions exhibited ripe fruit with greater firmness compared to those grown in field conditions. In fact, some panelists indicated that they noticed greater firmness for the ripe fruit harvested in protected conditions, which allowed them to differentiate it in the duo–trio test. The strawberry firmness significantly increased with increased electrical conductivity, from 1.5 to 4.5 mS cm^−1^ (D'Anna et al., 2003). In this way, in the future, it would be interesting to evaluate the effect of increasing conductivity in protected cultivation conditions since firmness is the crucial quality parameter in the raspberry market, defining its sale as fresh, frozen, or pulp fruit.

In the present study, the ripening process exhibited consistent changes in color parameters across both growing conditions, characterized by a reduction in brightness, hue angle, and yellow-blue coloration (b*), alongside increased redness (a*). This trend was particularly evident in transitioning from the large green (LG) to the pink (P) stages for both conditions (**Figure 2G**). However, during postharvest, the fruit of open field conditions was observed with a dark red color compared to protected conditions (**Figure 2**), being differentiated by a panelist (**Figure 6A**), an interesting result since markets prefer a brighter red color.

We revealed that the growing conditions significantly influence the total soluble solids content (TSS) of ripe fruit, compared to other less influencing factors such as titratable acidity (TA) in fruit. Open field cultivation resulted in a higher TSS at the ripe stage than in protected soilless culture, indicating that fruits grown in the open field may have a sweeter taste profile. Furthermore, the open field fruits maintained a higher TSS after postharvest storage and shelf life, suggesting better retention of sweetness over time, while TA levels remained consistent postharvest for both cultivation methods. These differences in soluble solids could be due to differences in irrigation, temperature, or radiation. Pritts et al. (1999) showed that raspberry growing in the greenhouse tended to be slightly less sweet and more acidic but well within the limits of acceptability. Previous reports on strawberry grown in soilless conditions showed a scarcely influenced by different electrical conductivity levels in the sugar content (D'Anna et al., 2003); however, this effect should be studied in raspberry. On the other hand, it has been reported that Heritage ripe raspberry from Alto do Rio Grande in Brazil, which experiences a temperate and semiarid subtropical climate, exhibited higher acidity and TSS than those from the cooler and wetter subtropical clime in the Mantiqueira Mountains. Conversely, the fruits of the Mantiqueira Mountains had a significantly higher total sugar content, although both localities produced fruits with comparable moisture levels (Maro et al., 2014). In the present study, the protected soilless culture provided a wider range of temperature (6.5°C–39°C) and relative humidity (23%–95%) (**Supplementary Table S1**) compared to open field conditions (11.7°C–20.4°C; 62–92%) during ripening (January 2022). Therefore, it is not easy to establish differences in the content of soluble solids due to temperature.

Ripe raspberry is mainly composed of glucose and fructose (Graham and Simpson, 2018), and fructose concentration is a crucial factor influencing the quality and marketability of raspberries (Kafkas et al., 2008; Dincheva et al., 2013). In the present study, the metabolomic analysis provides valuable insights into the complex metabolic changes occurring in raspberries during three stages of development (W, P, and R stages) and after postharvest storage of ripe fruit compared to two different growing conditions. The PCA discriminated the samples from both growing conditions (**Figure 3**). The fruit from Casablanca (open field) correlated with the main sugars of raspberry (D-glucose and D-fructose). In contrast, samples from Quillota (protected soilless culture) exhibited a positive correlation with all the amino acids detected by GC-MS, such as L-alanine, L-glutamic acid, glycine, L-serine, and L-valine. Durán-Soria et al. (2021) reported that fructose and glucose levels, together with citric acid (the main acid in raspberry fruits), were higher in raspberry fruits collected in Norway and Poland (the two coldest locations), which may, in part, be responsible for higher TSS and acidity. In the present study, the temperatures recorded in the protected culture are lower than the minimum temperatures reported for the open field, so the correlations observed are likely due to the differences between maximum temperatures. Moreover, a positive correlation between sucrose and fructose, and sucrose and glucose were reported by other authors (Dincheva et al., 2013; Akšić et al., 2022), which agrees with our findings, observing a positive correlation between these sugars (**Supplementary Figure 1**). Sensory evaluation confirmed the analytical results, with panelists able to discern sweetness differences, corroborating the significant variations in TSS and primary metabolites. These findings emphasize the influence of cultivation methods on the sensory and chemical properties of raspberries, which can guide growers in optimizing fruit quality for consumer preferences.

Citric and malic acid are the primary organic acids found in raspberries that contribute to the characteristic tartness of the fruit (Akšić et al., 2022). In our study, those organic acids did not show significant differences between both growing conditions when all stages were evaluated. Furthermore, the evolution of each metabolite during fruit developmental and postharvest was observed for both growing conditions through PLS-DA (**Figure 4**). At the pink stage, the metabolites that showed differences were D-gluconic acid and glucose-6-phosphate, which are related to the pentose phosphate pathway (PPP) and were more abundant in fruit grown in open field. The PPP serves as a respiratory pathway, playing a crucial role as a main supplier of NADPH in plants. It is closely linked to plant growth, development, and response to various environmental stresses (Tao et al., 2022). However, the main differences in primary metabolites were found in shelf-life stage, where the fruit from the open field besides showing higher sugar contents also showed lower contents of amino acids, malic acid, and α-ketoglutaric acid than the fruit from the protected soilless culture (**Figure 5B**). These metabolites are involved in important metabolic pathways such as the alanine, aspartate, and glutamate metabolism, and glycine, serine, and threonine metabolism, which showed a higher pathway impact during 1 day of shelf life at 20°C (**Figure 5A**). Rodríguez et al. (2019) reported that higher electrical conductivity of nutrient solution of tomatoes grown during winter showed a higher concentration of amino acids, which may be due to an imbalance in the metabolic processes associated with the flow of carbon and nitrogen. On the other hand, Ren et al. (2023) emphasized the role of amino acids such as L-lysine and L-glutamine in contributing to the antioxidant capacity of raspberries, which should be further studied for both conditions. Therefore, the relationship of the metabolites with essential pathways for plant growth and stress response emphasizes the influence of growing conditions on the metabolic profile and quality of raspberries.

## 5 Conclusions

Our results indicate that the protected soilless culture growing conditions are a good alternative for cultivating raspberries in areas with unsuitable soil and temperature fluctuations through the utilization of adjusted continuous fertigation to growing conditions with nutrient solutions controlled by electrical conductivity, achieving greater fruit weight and a potential firmness improvement. Although the lower content of soluble solids and a difference in the metabolites related to sugars were observed in the fruit of protected soilless conditions, which was perceived at the sensory level, these fruits show a better size and good evolution of color and weight during cold storage. The present study provides interesting and valuable results with a direct commercial application for this alternative growing system, mainly in areas where soil and water scarcity are a reality.

## Data availability statement

The original contributions presented in the study are included in the article/**Supplementary Material**. Further inquiries can be directed to the corresponding author.

## Ethics statement

The studies involving humans were approved by Comité de Bioseguridad, Bioética y Ética, Centro Regional de Estudios en Alimentos Saludables-CREAS. The studies were conducted in accordance with the local legislation and institutional requirements. The participants provided their written informed consent to participate in this study.

## Author contributions

CF: Conceptualization, Writing – review & editing, Formal analysis, Supervision. FÁ: Formal analysis, Methodology, Writing – review & editing. EP: Formal analysis, Methodology, Writing – review & editing. SV: Methodology, Writing – review & editing. MS: Formal analysis, Methodology, Writing – review & editing. DR: Formal analysis, Methodology, Writing – review & editing. AA: Formal analysis, Visualization, Writing – review & editing. JA: Conceptualization, Supervision, Writing – review & editing. MV: Conceptualization, Supervision, Writing – review & editing. CRF: Writing – review & editing. LF: Conceptualization, Methodology, Formal analysis, Writing – review & editing, Supervision, Project administration.

## Supplementary material

Supplementary Figure 1 Metabolite correlation. The correlation between metabolite compounds was carried out by the Pearson method using all data (fruit samples of developmental stages and ripe fruit postharvest).

## References

[B1] Akhatou I. González-Domínguez R. Fernández-Recamales Á. (2016). Investigation of the effect of genotype and agronomic conditions on metabolomic profiles of selected strawberry cultivars with different sensitivity to environmental stress. Plant Physiol. Biochem. 101, 14–22. doi: 10.1016/j.plaphy.2016.01.016 26841267

[B2] Akšić M. F. Nešović M. Ćirić I. Tešić Ž. Pezo L. Tosti T.. (2022). Chemical fruit profiles of different raspberry cultivars grown in specific Norwegian agroclimatic conditions. Horticulturae 8, 765. doi: 10.3390/horticulturae8090765

[B4] Álvarez F. Moya M. Rivera-Mora C. Zúñiga P. E. Jara-Cornejo K. Muñoz P.. (2023). Abscisic acid synthesis and signaling during the ripening of raspberry (*Rubus idaeus* ‘Heritage’) fruit. Plants 12, 1882. doi: 10.3390/plants12091882 37176940 PMC10180958

[B5] Anjos R. Cosme F. Gonçalves A. Nunes F. Vilela A. Pinto T. (2020). Effect of agricultural practices, conventional vs organic, on the phytochemical composition of ‘Kweli’ and ‘Tulameen’ raspberries (*Rubus idaeus* L.). Food Chem. 328, 126833. doi: 10.1016/j.foodchem.2020.126833 32480265

[B3] AOAC (Association of Official Analytical Chemists) (1997). Official Methods of Analysis of AOAC International. 16th ed (Washington, DC, USA: Association of Official Analytical Chemists Inc).

[B6] Balawejder M. Matłok N. Piechowiak T. Szostek M. Kapusta I. Niemiec M.. (2023). The modification of substrate in the soilless cultivation of raspberries (*Rubus idaeus* l.) as a factor stimulating the biosynthesis of selected bioactive compounds in fruits. Molecules 28, 118. doi: 10.3390/molecules28010118 PMC9822297 36615315

[B1001] Bernales M. Monsalve L. Ayala-Raso A. Valdenegro M. Martínez J. P. Travisany D.. (2019). Expression of two indole-3-acetic acid (IAA)-amido synthetases (GH3) genes during fruit development and ripening of raspberry (Rubus idaeus Heritage). Sci. Hortic. 246, 168–175. doi: 10.1016/j.scienta.2018.09.077

[B1002] Contreras C. Hermosilla A. Contreras E. Naranjo P. Zoffoli J.P. Gambardella M.. (2021). Postharvest physiology and storage potential of new Chilean raspberry cultivars. Chil. J. Agric. Res. 81 (2), 161–171. doi: 10.4067/S0718-58392021000200161

[B7] D'Anna F. Incalcaterra G. Moncada A. Miceli A. (2003). Effects of different electrical conductivity levels on strawberry grown in soilless culture. Acta Hortic. 609, 355–360. doi: 10.17660/ActaHortic.2003.609.53

[B8] Dincheva I. Badjakov I. Kondakova V. Batchvarova R. (2013). Metabolic profiling of red raspberry (*Rubus idaeus*) during fruit development and ripening. Int. J. Agric. Sci. Res. 3, 81–88.

[B9] Durán-Soria S. Pott D. M. Will F. Mesa-Marín J. Lewandowski M. Celejewska K.. (2021). Exploring genotype-by-environment interactions of chemical composition of raspberry by using a metabolomics approach. Metabolites 11, 490. doi: 10.3390/metabo11080490 34436431 PMC8398420

[B10] Frías-Moreno M. N. Parra-Quezada R. A. González-Aguilar G. Ruíz-Canizales J. Molina-Corral F. J. Sepulveda D. R.. (2021). Quality, bioactive compounds, antioxidant capacity, and enzymes of raspberries at different maturity stages, effects of organic vs. Conventional fertilization. Foods 10, 953. doi: 10.3390/foods10050953 33925426 PMC8145102

[B11] Fuentealba C. Hernández I. Saa S. Toledo L. Burdiles P. Chirinos R.. (2017). Colour and in *vitro* quality attributes of walnuts from different growing conditions correlate with key precursors of primary and secondary metabolism. Food Chem. 232, 664–672. doi: 10.1016/j.foodchem.2017.04.029 28490125

[B1003] Fuentes L. Monsalve L. Morales-Quintana L. Valdenegro M. Martínez J.P. Defilippi B.G.. (2015). Differential expression of ethylene biosynthesis genes in drupelets and receptacle of raspberry (Rubus idaeus). J. Plant Physiol. 179, 100–105. doi: 10.1016/j.jplph.2015.02.005 25847526

[B12] Graham J. Simpson C. (2018). “Developmental transitions to fruiting in red raspberry,” in The genomes of rosaceous berries and their wild relatives. Compendium of Plant Genomes. Eds. Hytönen T. Graham J. Harrison R. (Cham: Springer), 199–212.

[B1004] Hirzel J. (2013). “Fertilización de la frambuesa.” in Manual de frambuesa. Eds. Undurraga P. Vargas e I. (Santiago, Chile: Boletín INIA N°264).

[B13] Iborra-Bernad C. Tárrega A. García-Segovia P. Martínez-Monzó J. (2014). Comparison of vacuum treatments and traditional cooking using instrumental and sensory analysis. Food analytical Methods 7, 400–408. doi: 10.1007/s12161-013-9638-0

[B14] ISO (2005). Sensory analysis. Methodology. Paired comparison test. Standard No. 5495 (Geneva, Switzerland: International Organization for Standardization).

[B15] Kafkas E. Özgen M. Özoğul Y. Türemiş N. (2008). Phytochemical and fatty acid profile of selected red raspberry cultivars: A comparative study. J. Food Qual. 31 (1), 67–78. doi: 10.1111/j.1745-4557.2007.00184.x

[B16] Kassim A. Poette J. Paterson A. Zait D. McCallum S. Woodhead M.. (2009). Environmental and seasonal influences on red raspberry anthocyanin antioxidant contents and identification of quantitative traits loci (QTL). Mol. Nutr. Food Res. 53, 625–634. doi: 10.1002/mnfr.200800174 19156716

[B17] Kotuła M. Kapusta-Duch J. Smoleń S. Doskočil I. (2022). Phytochemical composition of the fruits and leaves of raspberries (*Rubus idaeus* l.)—conventional vs. organic and those wild grown. Appl. Sci. 12, 11783. doi: 10.3390/app122211783

[B19] Marchi P. M. Carvalho I. R. Pereira I. da Rosa T. C. Höhn D. Szareski V. J.. (2019). Yield and quality of primocane-fruiting raspberry grown under plastic cover in southern Brazil. Sci. Agric. 76 (6), 481–486. doi: 10.1590/1678-992x-2018-0154

[B18] Maro L. A. C. Pio R. Guedes M. N. S. Abreu C. M. P. D. Moura P. H. A. (2014). Environmental and genetic variation in the post-harvest quality of raspberries in subtropical areas in Brazil. Acta Scientiarum. Agron. 36, 323–328. doi: 10.4025/actasciagron.v36i3.18050

[B20] Mihafu F. D. Issa J. Y. Kamiyango M. W. (2020). Implication of sensory evaluation and quality assessment in food product development: A review. Curr. Res. Nutr. Food Sci. J. 8 (3), 690–702. doi: 10.12944/CRNFSJ.8.3.03

[B21] Monsalve L. Bernales M. Ayala-Raso A. Álvarez F. Valdenegro M. Alvaro J.-E.. (2022). Relationship between endogenous ethylene production and firmness during the ripening and cold storage of raspberry (*Rubus idaeus* ‘Heritage’) fruit. Horticulturae 8, 262. doi: 10.3390/horticulturae8030262

[B22] Moya C. Oyanedel E. Verdugo G. Flores M. F. Urrestarazu M. Álvaro J. E. (2017). Increased electrical conductivity in nutrient solution management enhances dietary and organoleptic qualities in soilless culture tomato. Hortic. Sci. 52 (6), 868–872. doi: 10.21273/HORTSCI12026-17

[B1005] Perkins-Veazie P. Nonnecke G. (1992). Physiological changes during ripening of raspberry fruit. HortSci. 27 (4), 331–333. doi: 10.21273/HORTSCI.27.4.331

[B23] PN-ISO 3972:2016–07 (2016). Analiza sensoryczna – Metodyka – Metody badania wrażliwości smakowej (Sensory analysis – Methodology – Method of investigating sensitivity of taste). (Katowice, Poland: Polski Komitet Normalizacyjny (Polish Committee for Standardization)). Available at: https://sklep.pkn.pl/pn-iso-3972-2016-07p.html.

[B24] Popović T. Šarić B. Martačić J. D. Arsić A. Jovanov P. Stokić E.. (2022). Potential health benefits of blueberry and raspberry pomace as functional food ingredients: Dietetic intervention study on healthy women volunteers. Front. Nutr. 9, 969996. doi: 10.3389/fnut.2022.969996 36061889 PMC9428553

[B25] Pritts M. P. Langhans R. W. Whitlow T. H. Kelly M. J. Roberts A. (1999). Winter raspberry production in greenhouses. HortTechnology 9 (1), 13–15. doi: 10.21273/HORTTECH.9.1.13

[B26] Quezada C. Soriano M. A. Díaz J. Merino R. Chandía A. Campos J.. (2014). Influence of soil physical properties on grapevine yield and maturity components in an ultic palexeralf soils, Central-Southern, Chile. Open J. Soil Sci. 4 (04), 127. doi: 10.4236/ojss.2014.44016

[B27] Red Agrometereológica INIA data. Available at: https://agrometeorologia.cl/ (Accessed 12 October 2023).

[B28] Ren X. Wang S. Wang J. Xu D. Ye Y. Song Y. (2023). Widely targeted metabolome profiling of different plateau raspberries and berry parts provides innovative insight into their antioxidant activities. Front. Plant Sci. 14, 1143439. doi: 10.3389/fpls.2023.1143439 36993862 PMC10042140

[B29] Rodríguez F. Pedreschi R. Fuentealba C. de Kartzow A. Olaeta J. A. Alvaro J. E. (2019). The increase in electrical conductivity of nutrient solution enhances compositional and sensory properties of tomato fruit cv. Patrón. Scientia Hortic. 244, 388–398. doi: 10.1016/j.scienta.2018.09.059

[B30] Stewart D. Iannetta P. P. Davies H. V. (2001). Ripening-related changes in raspberry cell wall composition and structure. Phytochemistry 56, 423–428. doi: 10.1016/S0031-9422(00)00410-6 11261574

[B31] Stojanov D. Milošević T. Mašković P. Milošević N. Glišić I. Paunović G. (2019). Influence of organic, organo-mineral and mineral fertilisers on cane traits, productivity and berry quality of red raspberry (*Rubus idaeus* L.). Sci. Hortic. 252, 370–378. doi: 10.1016/j.scienta.2019.04.009

[B32] Tao S. Zhu Y. Pan Y. Zhang Z. Huang L. (2022). Enhancement of respiratory metabolism of the pentose phosphate pathway (PPP) strengthens the chilling tolerance of postharvest papaya fruit stored at 1 °C. Postharvest Biol. Technol. 91, 111988. doi: 10.1016/j.postharvbio.2022.111988

[B33] Vicente A. Ortungo C. Powell A. Greve L. Labavitch J. (2007). Temporal sequence of cell wall disassembly events in developing fruits. 1. Analysis of raspberry (*Rubus idaeus*). J. Agric. Food Chem. 55, 4119–4124. doi: 10.1021/jf063547r 17428067

