# Supplementary figures and images for: Differences in primary metabolism related to quality of raspberry (Rubus idaeus L.) fruit under open field and protected soilless culture growing conditions
Source: Front Plant Sci. 2024 Jan 11;14:1324066. doi: 10.3389/fpls.2023.1324066 (PMC10808700; doi:10.3389/fpls.2023.1324066)

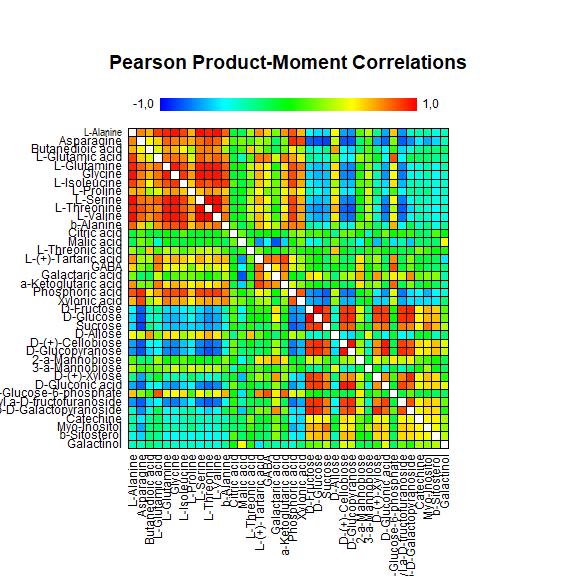

Supplement: Supplementary Figure 1 — Metabolite correlation. The correlation between metabolite compounds was carried out by the Pearson method using all data (fruit samples of developmental stages and ripe fruit postharvest). [file Image_1.jpeg]
