# Supplementary material for: Differences in primary metabolism related to quality of raspberry (Rubus idaeus L.) fruit under open field and protected soilless culture growing conditions
Source: Front Plant Sci. 2024 Jan 11;14:1324066. doi: 10.3389/fpls.2023.1324066 (PMC10808700; doi:10.3389/fpls.2023.1324066)
Supplement: Supplementary file 2 [file Table_1.docx]

Table S1. Temperature and humidity register in open field and protected soilless condition.

|  | **Minimum air temperature** | **Maximum air temperature** | **Minimum relative humidity** | **Maximum relative humidity** |
| --- | --- | --- | --- | --- |
| Open field condition* | 11.7 | 20.4 | 62 | 92 |
| Protected soilless condition** | 6.5 | 39.0 | 23 | 96 |

(*) Data downloaded from Red Agrometereológica INIA data (<https://agrometeorologia.cl/>).

(**) Data registered using a Temperature/Relative Humidity Recorder.
